# Design and Parameter Research of Time-Harmonic Magnetic Field Sensor Based on PDMS in Microfluidic Technology

**DOI:** 10.3390/polym12092022

**Published:** 2020-09-04

**Authors:** Chenzhao Bai, Hongpeng Zhang, Chengjie Wang, Lebile Ilerioluwa Joseph, Qiang Wang, Yucai Xie, Guobin Li

**Affiliations:** School of Marine Engineering, Dalian Maritime University, Dalian 116026, China; baichenz@dlmu.edu.cn (C.B.); wangcj@dlmu.edu.cn (C.W.); lebilei7@gmail.com (L.I.J.); xmwq2008@126.com (Q.W.); xyc86418332@dlmu.edu.cn (Y.X.); guobinli88@163.com (G.L.)

**Keywords:** solenoid magnetic field, coil parameter, microfluidic technology, PDMS application

## Abstract

In order to improve the throughput and sensitivity of the inductive metal micro-abrasive particle detection sensor, this paper uses microfluidic detection technology to design a high-throughput abrasive particle detection sensor based on PDMS (Polydimethylsiloxane). Theoretical modeling analyzes the magnetization of metal abrasive particles in the coil’s time-harmonic magnetic field, and uses COMSOL simulation to calculate the best performance parameters of the sensor. Through the experiment of the control variable method, the corresponding signal value is obtained and the signal-to-noise ratio (SNR) is calculated. The SNR value and error value are calculated, and the SNR is corrected. The detection limit of the sensor is determined to be 10 μm iron particles and 60 μm copper particles. The optimal design parameters of the 3-D solenoid coil and the frequency characteristics of the sensor are obtained. Finally, through high-throughput experiments and analysis, it was found that there was a reasonable error between the actual throughput and the theoretical throughput. The design ideas suggested in this article can not only improve the sample throughput, but also ensure the detection accuracy. This provides a new idea for the development of an inductive on-line detection method of abrasive particle technology.

## 1. Introduction

Hydraulic systems and lubrication systems are the “blood” of mechanical equipment, which are indispensable components of the equipment. Wear is one of the main factors affecting the reliability and service life of mechanical equipment [1]. The hydraulic oil and lubricating oil contain a wealth of information on mechanical wear. This is because the wear debris generated by the friction pair is collected in the oil system and circulated within the oil [2]. Therefore, the detection of wear debris is of great significance for online recognition of abnormal wear conditions and prediction of the life span of the equipment.

For equipment wear diagnosis, there are historical technologies, but now more development is being made towards online diagnosis technology. According to different principles, there are several monitoring methods, including mechanical vibration, spectrum, oil analysis and other technologies [3]. The oil analysis method can directly reflect the wear status of machinery and other information, and it is widely used in online monitoring. It includes: optical detection [4], ultrasonic detection [5,6], capacitance detection [7,8] and inductance detection [9,10,11]. Among these methods, inductive detection is most used for on-line monitoring of wear debris. This is due to its outstanding advantages, such as simple structure, convenient processing, easy handling and good stability; it can distinguish ferromagnetic and non-ferromagnetic metal fragments during detection [12,13,14].

PDMS is widely used in the field of microfluidics, and oil detection technology is also developed in this field. This technology mainly uses PDMS as the matrix to produce chips that can use inductive technology to identify wear particles in the oil. The main principle of the inductive method was developed by applying Faraday’s law of electromagnetic induction. When a high-frequency alternating current is applied to the induction coil, an alternating electromagnetic field is generated, and the wear debris is magnetized in the alternating electromagnetic field [15,16]. The inductance detection method is used to obtain information by diagnosing the pulse signal generated by the change of the magnetic field after the wear debris is magnetized. There are different types of inductive sensors due to differences in the structure of their induction coil, but they can be divided into two types: plane coil type and 3-D solenoid type [17,18]. 

Du et al. [19] developed a four-channel sensor based on the structure of the planar coil, which can collect the voltage signal value of 55–70 μm iron particles and increase the detection throughput. Ma et al. [20] designed a high throughput inductive metal detector using inductive planar coils. The iron core is added to the detector, which can identify 40 μm iron and 90 μm copper particles. Zeng et al. [21] designed a multi-pollutant detector using double-plane coils, which can not only detect metal pollutants using inductance, but also developed a capacitor to detect water droplets and bubbles in oil. However, due to the complex structure, its detection accuracy is very low. Yin et al. [22] conducted in-depth research on metal detectors with planar coil structure, and analyzed that metal particles at different positions of the planar coil would have different changes in the detection results, which provided a basis for the design of this type of detector. Based on the planar coil, Timur et al. [23] created an inductive sensor through the Sprott chaotic oscillator and a planar printed circuit board inductor coil, and proposed a new method of real-time oscillation analysis using a band-pass filter. This technology helps metal detectors have improved data accuracy. The above is the relatively new technology of the planar inductance coil metal detector. The planar inductance coil has a simple and stable structure with less diversity. For metal particles, detection accuracy is low, and the detection effect needs to be improved.

Metal detectors with 3-D solenoid structure have been developed earlier, and there are already relatively mature technologies and equipment. At present, the research on this type of metal detector mainly focuses on the detection accuracy, detection throughput and sensor stability. Du et al. [24] wound a solenoid coil on a capillary glass tube and used LCR precision meters to successfully distinguish between 50–75 μm iron particles and 100–125 μm copper particles. Although this technology improves the detection accuracy, the detection throughput is low. Feng et al. [25] proposed a new inductance detector structure, which has multiple sensing coils inside an excitation coil, which can detect 120 μm iron particles and 210 μm copper particles in a pipe with diameter of 34 mm. Then they designed a three-coil solenoid structure [26] that uses the non-uniform magnetic field generated by the coil to identify iron particles of 134 μm and copper particles of 230 μm in a 43 mm pipe. They increased the detection throughput, but reduced the detection accuracy. Le et al. [27] also designed a three-coil structure metal detector applied to the gearbox of a wind turbine. This system can effectively detect metal abrasive particles of 200 μm. In order to improve the detection accuracy of the inductive metal detector, Zhang et al. [28] invented a processing method with the “0” distance between the channel and the 3-D solenoid coil. This method can greatly improve the detection accuracy of particles, and it can detect 50 μm iron particles and 120 μm copper particles. Next, Zeng et al. [29] designed a 3-D solenoid structure with silicon steel sheet, which uses the high magnetic permeability of silicon steel sheet to concentrate the coil magnetic field in the detection area, and can detect 18 μm iron particles and 75 μm Copper particles. The above are relatively new technologies of 3-D solenoid metal detectors, although there are many types, and they all have their own advantages. Some technologies have high detection accuracy, but low detection throughput. If the detection throughput is increased, the detection accuracy is reduced.

**Figure 1:**
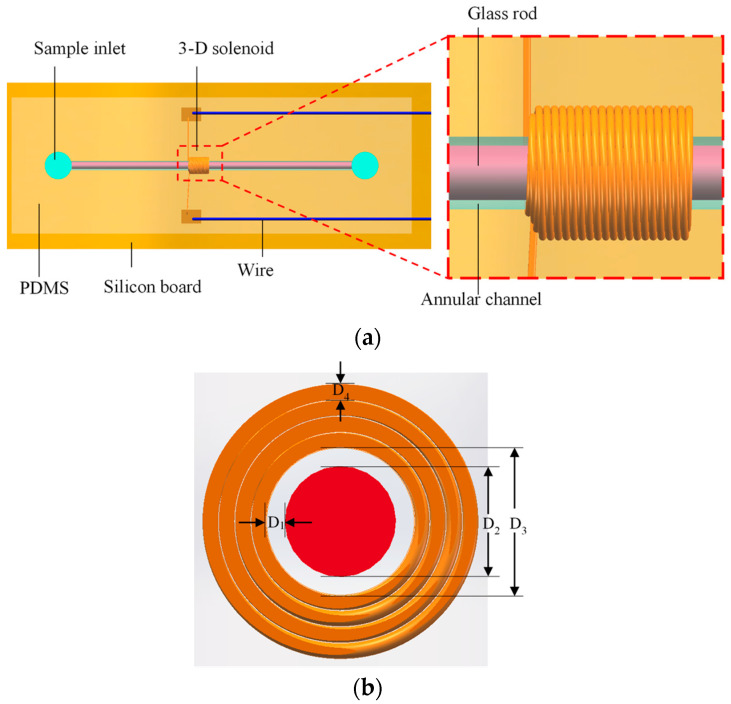
(**a**) The overall design of the sensor; (**b**) 3-D solenoid cross-section. The diameter of the annular channel D_1_ = 300 μm; the diameter of the glass rod D_2_ = 1200 μm; the overall diameter of the channel D_3_ = 1800 μm; the diameter of the solenoid D_4_ = 30 μm.

In order to solve the problems of detection accuracy and detection throughput at the same time, our team has been committed to research in this area. Based on the 3-D solenoid coil, this paper designs a special annular channel belonging to the 3-D solenoid, as shown in Figure 1. This annular channel not only improves the detection flux of the channel, but also takes advantage of the 3-D solenoid coil itself. All the magnetic fields generated by the solenoid coil are used to detect metal abrasive, which greatly improves the detection efficiency of this type of coil. The following is the design and experimental study of the structure.

## 2. PDMS-Based Chip Design and Fabrication 

As shown in Figure 1, (a) is the overall design of the sensor. The annular microchannel passes through the inner hole of the 3-D solenoid coil, and the two ends of the solenoid coil are connected to the connection line. (b) is a 3-D solenoid section, D_1_ is the diameter of the annular channel; D_2_ is the diameter of the glass rod built into the annular channel; D_3_ is the overall diameter of the channel; D_4_ is the diameter of the 3-D solenoid.

Manufacturing process: First, a 3-D solenoid coil is wound using a precision winding machine (Shi Li SRDZ23-1B, Zhong Shan Shi Li Wire Winder Equipment, Zhong Shan, China) and the solenoid coil is wound on a copper rod with a diameter of 1800 μm. The wound 3-D solenoid is then fixed with a copper rod to the correct position of the silicon board. The wires are connected and debugged; Next, PDMS is mixed with the curing agent at a ratio of 10:1, and then the model is cast. After 1 h, the 1800 μm copper rod is pulled out, the 1200 μm glass rod is fixed in the correct position inside the channel, and the channel opening is sealed. Finally, samples for import and export are made and the production is completed.

## 3. Theoretical Analysis

**Figure 2:**
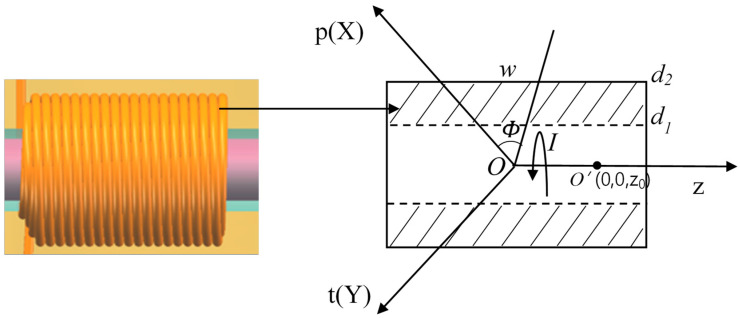
Solenoid coil modeling.

The detection principle is shown in the figure. A single solenoid coil with an alternating current of *I* is equivalent to a hollow cylindrical inductor, and the current direction is shown in the figure. The cylindrical coordinate system (Ρ,Φ,Ζ) is established with the center of the cross-section circle as the origin, and the Ζ axis is taken as its central axis. The length of the hollow cylindrical coil is w, the inner diameter is $d_{1}$, and the outer diameter is $d_{2}$. After the coil is energized, it will generate a closed electromagnetic field in the spatial range, which is assumed to be a magnetic field uniformly distributed in space-time. According to the general impedance expression of the coil [30]:

(1) $$Z=\;-\frac{1}{I^{2}}\int_{\Omega }E\cdot J_{c}d\Omega Z$$

(2) $$J_{c}=n_{c}I{\hat{J}}_{c}$$

where I is the current in the solenoid coil, $n_{c}$ is the current’s turn density,$\;J_{c}$ is the complex current density, E is the complex electric field and Ω is the space occupied by the solenoid coil in space. It is assumed that the passage of metal particles in the coil will only cause the impedance change of the coil and will not affect the distribution of the space-time magnetic field outside the coil. When metal particles pass at any point $O^{\prime }\left(0,0,z_{0}\right)$ on the coil axis (as shown in Figure 2), according to the Biot-Savart theorem, the magnetic field distribution $B_{z}$ affected by the metal particles can be expressed as [31]:

(3) $$B_{z}=\frac{\mu_{0}n_{c}I}{2}\int_{\Omega }\rho^{2}{\left[\rho^{2}+{\left(z-z_{0}\right)}^{2}\right]}^{-3/2}d\Omega$$

The magnetic vector potential of the particles is:

(4) $$\Delta A=B_{z}K_{p}{\left[\rho^{2}+{\left(z-z_{0}\right)}^{2}\right]}^{-3/2}$$

Define $\int_{\Omega }\rho^{2}{\left[\rho^{2}+{\left(z-z_{0}\right)}^{2}\right]}^{-3/2}d\Omega$ as the geometric feature of the coil and the position function of the particle $P(z_{0})$:

(5) $$P(z_{0})=\int_{\Omega }\rho^{2}{\left[\rho^{2}+{\left(z-z_{0}\right)}^{2}\right]}^{-3/2}d\Omega$$

The magnetization model of metal particles in the space-time uniform magnetic field is an axisymmetric model, so the impedance change of the solenoid coil affected by the particles can be expressed as:

(6) $$\Delta Z=\frac{j\omega n_{c}}{I}\int_{\Omega }\Delta Ad\Omega$$

Substituting Equations (2)–(4) into (6), when the metal particles pass along the axis of the solenoid coil, the change in impedance of the coil is:

(7) $$\Delta Z=j\pi u_{0}n_{c}^{2}K_{p}P{\left(z_{0}\right)}^{2}$$

For the same kind of particles, the magnetization factor $K_{p}$ is only related to the particles themselves, and the same kind of coil $n_{c}$ is only related to the winding method of the coil. When exploring the changing rules of different inner diameter, length and other parameters of the coil $\left(P{\left(z_{0}\right)}^{2}\right)$, the peak value of impedance change will appear on the coil interruption surface. Therefore, substituting $z_{0}=0$ into Equation (7) can get $\Delta Z_{max}$. The real part of $\Delta Z_{max}$ is the effect of particles on the chip resistance, and the imaginary part is the effect of particles on the inductance of the coil. The inductive formula can be obtained by the imaginary part of $\Delta Z_{max}$ as follows:

(8) $$\Delta L=Im\left(\frac{\Delta Z_{max}}{\omega }\right)=\frac{4\pi \mu_{0}N^{2}}{w^{2}+{d_{1}}^{2}}Re\left(K_{p}\right)$$

For ferromagnetic metal particles:

(9) $$K_{p}=\frac{r^{3}}{2}\cdot \frac{\left(-r^{2}k^{2}+2\mu_{r}+1\right)sin(rk)-rk\left(2\mu_{r}+1\right)cos(rk)}{\left(r^{2}k^{2}+\mu_{r}-1\right)sin(rk)-rk\left(\mu_{r}-1\right)cos(rk)}$$

For non-ferromagnetic metal particles, $\mu_{r}\approx 1$, which can be obtained by substituting into (9):

(10) $$K_{p}=-\frac{1}{2}\left[r^{3}+\frac{3r^{2}}{k}cot(rk)-\frac{3r}{k^{2}}\right]$$

In Formulas (9) and (10), r is the radius of the spherical ferromagnetic metal particles, $\mu_{r}$ is the relative permeability of the ferromagnetic metal particles, and the expression of k is:

(11) $$k=\sqrt{-j\omega \mu_{r}\mu_{0}\sigma }$$

In the formula, $j^{2}=-1$, ω is the angular frequency of the excitation power supply and σ is the conductivity of different particles.

**Figure 3:**
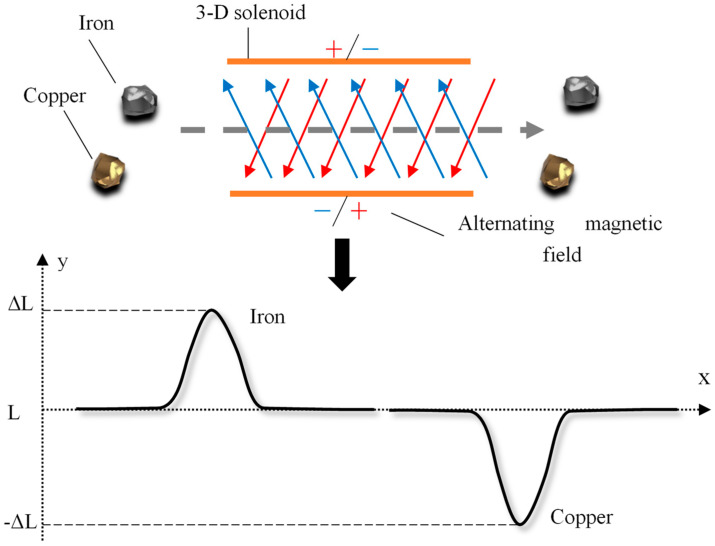
A 3-D solenoid coil detection principle diagram: When iron particles flow through the magnetic field area, *ΔL* > 0, the detection signal direction is upward, and when copper particles flow through the magnetic field area, *ΔL* < 0, the detection signal is downward.

Therefore, for the micro-inductance coil with high-frequency excitation alternating current, when the ferromagnetic metal particles or non-ferromagnetic metal particles mixed in the hydraulic oil pass through the magnetic field area generated by the inductance coil, these two metal particles will instantly be magnetized by a magnetic field. The magnetized ferromagnetic metal particles generate a new magnetic field under the action of electromagnetic induction. At the same time, they also generate eddy currents in the opposite direction to the original alternating magnetic field. As a result of their strong magnetic permeability, the new magnetic field strength is greater than the eddy current magnetic field strength. This will cause the magnetic flux of the micro-planar coil to increase, which in turn will increase the inductance at both ends of the inductive coil, *ΔL* is a positive value (*ΔL* > 0), as shown in Figure 3. In contrast, when non-ferromagnetic metal particles (such as copper particle contaminants) pass through the detection area, no magnetization occurs. The eddy current generated inside the particles cancels out part of the magnetic flux of the coil, reducing the coil inductance and *ΔL* is negative (*ΔL* < 0). Therefore, the nature and size of the metal particles can be judged by the change in the positive and negative size of *ΔL*. In addition to the *ΔL* value being affected by the properties and size of the particles, the detection inductance value *ΔL* is mainly related to the number of turns parameter and relative position. In the next section, the number of turns of the coil is mainly analyzed.

## 4. Simulation and Experimental Discussion

### 4.1. Simulation Analysis

**Figure 4:**
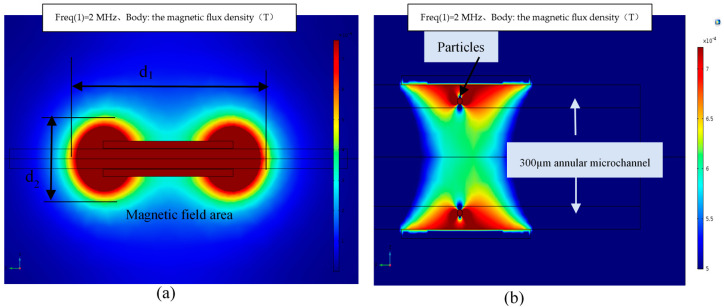

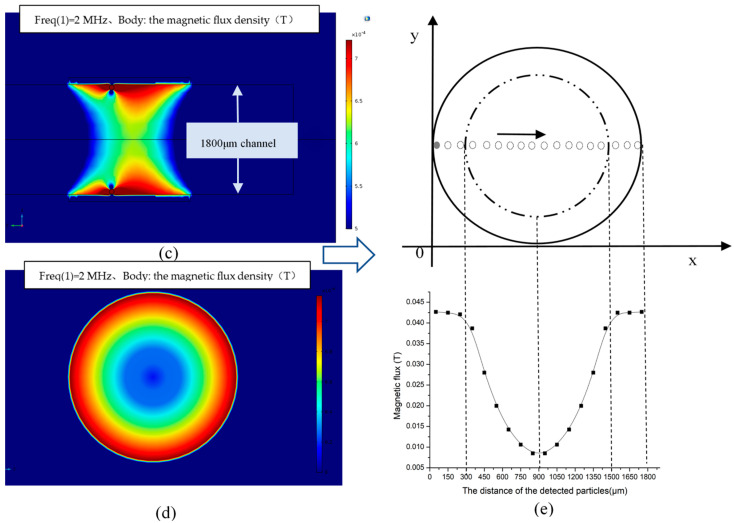
Analysis of COMSOL simulation results. (**a**) Cloud diagram of the magnetic field area of 300 μm microchannel, d1, d2 are the length and width of the magnetic field area; (**b**) cloud image of the simulation effect of iron particles in the 300 μm annular microchannel; (**c**) cloud image of the simulation effect of iron particles in the 1800 μm channel; (**d**) the section of the magnetic field inside the 1800 μm channel; (**e**) simulation results of the magnetic flux density of iron particles at different positions in the channel.

The COMSOL 5.3 Multiphysics software (COMSOL Inc., Stockholm, Sweden) was used to perform two-dimensional and three-dimensional simulation analysis of the sensor, and the parameter design was consistent with the actual sensor value. The frequency was 2 MHz, the voltage was 2 V, the inner diameter of the ring channel was 300 μm and the presented number of coil turns was 100 turns. The simulation analysis comparison between the annular microchannel and the 300 μm microchannel in other works [32] are established, as shown in Figure 4 below.

Figure 4 shows the simulation analysis results. (a) The 300 μm microchannel was designed by our team in the early stage. It has a low flux, but greatly improves the sensitivity. From the comparison of simulation results, the magnetic field distribution *d*_1_ and *d*_2_ in Figure 4, (a) are uneven, the length and width of the magnetic field area are too large, and there is an interfering magnetic field in the external area of the microchannel, which will affect the detection. The distribution of the magnetic field in (b) is more reasonable. In the annular shaped microchannel, the magnetic field is more concentrated on a certain part, and the magnetic field is not dispersed. (c) In the 1800 μm super-throughput channel, the channel has the largest throughput, but the detection accuracy is too low, and the pollutants will have different detection results at different positions of the channel. As shown in (e), the magnetic flux of the iron particles is the lowest when it is near the center of the channel, and the highest when it is near the edge, especially in the area of the annular shaped microchannel. The detection accuracy is high, and the result is close. (d) is the internal magnetic field section of the 1800 μm channel. The design of the annular shaped microchannel is more reasonable, and the magnetic field distribution has a greater advantage.

### 4.2. Discussion of 3-D Solenoid Coils Turns

**Figure 5:**
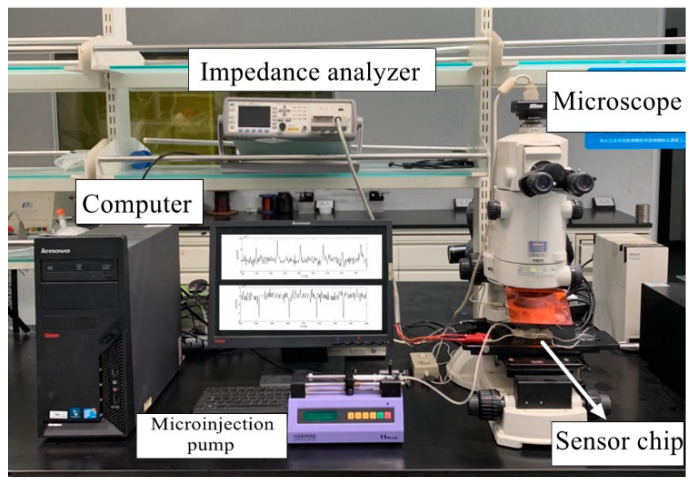
Photograph of the experimental system set-up.

The instruments used in the experiment included an impedance analyzer (Agilent E4980A, Agilent Technologies Inc., Bayan Lepas, Malaysia), a microscope (Nikon AZ100, Nikon, Tokyo, Japan), a microinjection pump (Harvard Apparatus B-85259, Harvard Apparatus, Holliston, MA, USA), a computer with a LabVIEW data acquisition unit, and our 3-D solenoid sensor. The experimental instruments were connected before the experiments, and the test bench system is shown in Figure 5.

**Figure 6:**
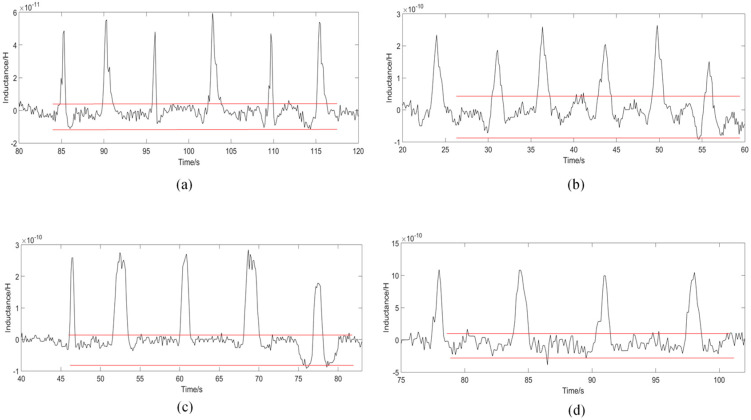

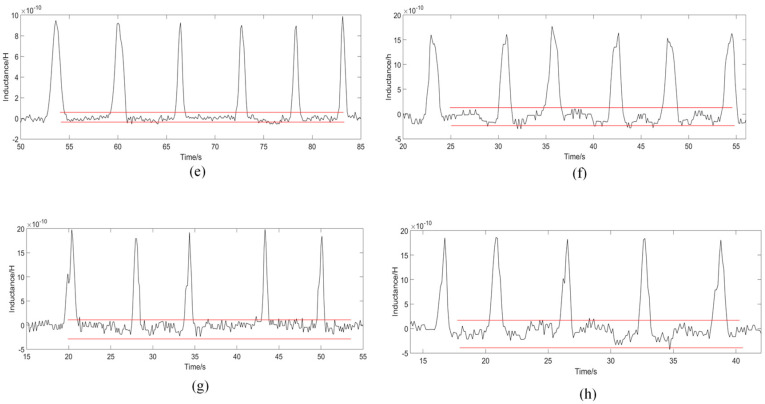
The detection signal value of different solenoid coils on 40 μm iron particles, (**a**–**h**) is 100~450 turns.

Analysis of the number of turns of the 3-D solenoid can further improve the detection performance of the sensor. The winding machine winds 3-D solenoids with 100, 150, 200, 250, 300, 350 and 400 turns. In this experiment, corresponding sensors were made based on these solenoids and turns analysis was performed. The iron particles and copper particles used in the experiments were customized by professional experimental equipment manufacturers (Beijing Huafeng Experimental Materials Co., Ltd., Beijing, China). In order to ensure the accuracy of the experimental data, 40 μm iron particles and 90 μm copper particles were used in the experiment. The particles were weighed to 5 mg separately, and mixed with 200 mL of hydraulic oil. The mixed oil was placed in an ultrasonicator (IKA S25, IKA Inc., Staufen, Germany) and shaken for 2 min, then put into the micro-injection pump and left for the experimental operation. The analysis of the experimental data used the method of multiple measurements and the average value. During the experiment, the impedance analyzer provided a voltage of 2 V, a frequency of 2 MHz, and a flow rate of the micro-injection pump of 600 μL/min. To ensure the accuracy of the experiment, each group of data was measured more than five times. The experimental results on the number of turns of the solenoid coil are shown in Figure 6 below.

According to Figure 6, as the number of turns of the coil increases, the value of the detection signal of the particles gradually increases, because the larger the number of turns of the coil, the stronger the magnetic field provided. When the solenoid coil is 100 turns, the average signal of 40 μm iron particles is 5.312 × 10^−11^. When the coil turns reach 450 turns, the detection signal is 1.895 × 10^−9^. However, as the number of turns increases, the detection signal increases, and the detected noise value also changes. At 100 turns, the average noise value is 1.302 × 10^−11^. At 300 turns detection, the average noise is 8.150 × 10^−11^. At 450 turns, the average noise value increases to 3.907 × 10^−10^. According to the results, from 100 to 300 turns, the noise value increases first and then decreases; from 300 to 450 turns, the noise value increases rapidly. So, after calculation, the noise value of the coil is the lowest at 300 turns, which shows that the noise has the smallest impact on the signal currently.

For inductive detection sensors, the level of sensitivity can be expressed by the signal-to-noise ratio (SNR), which represents the detection capability of the sensor. The larger the SNR value, the stronger the detection sensitivity. The SNR calculation formula is:

(12) $$SNR=\frac{Signal\;value}{Noise\;value}$$

In the 100–300 turns coil, the noise value of the particle detection is relatively low, but as the number of turns increases, the detection signal increases, and the noise value also increases, but the speed of the signal increase currently is faster than the speed of noise increase. When the coil is 300 turns, the ratio of signal to noise reaches the maximum. When the number of coil turns is greater than 300 turns, the speed of noise increase is significantly accelerated, and the speed of signal value increase gradually becomes slower, which results in a decrease in the SNR. As shown in Figure 8a, the signal value and SNR value of 40μm iron particles in the coil of 100–450 turns.

**Figure 7:**
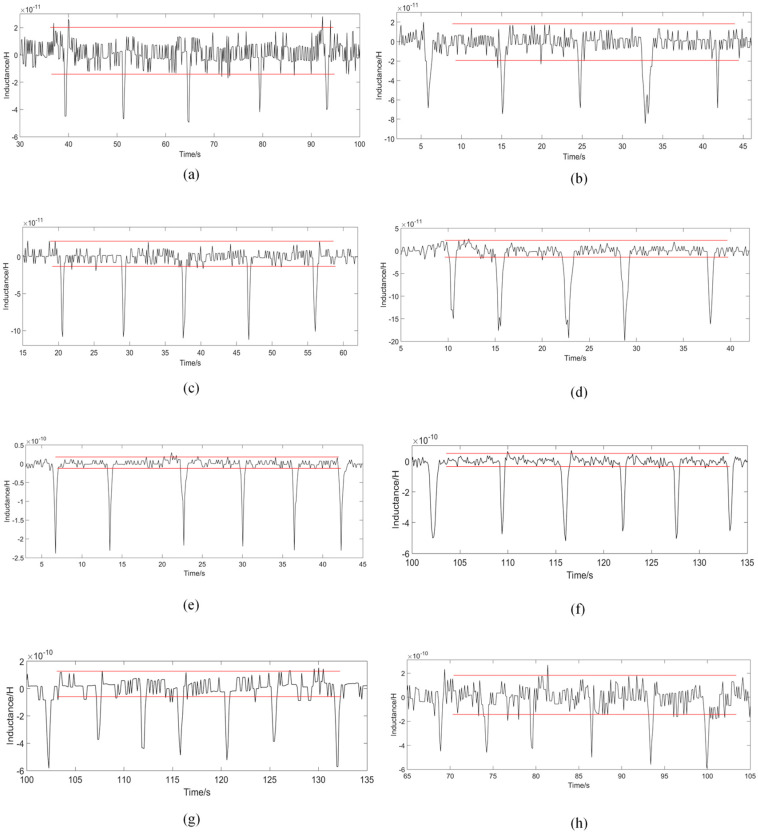
The detection signal value of different solenoid coils on 90 μm copper particles, (**a**–**h**) is 100~450 turns.

The copper particles are detected in the same way. The impedance analyzer provides a voltage of 2 V, a frequency of 2 MHz at the maximum frequency, and a flow rate of the micro-injection pump of 800 μL/min. Each group of experimental data is measured more than five times for correction. The experimental results regarding the number of coils turns on copper particles are shown in Figure 7 below.

**Figure 8:**
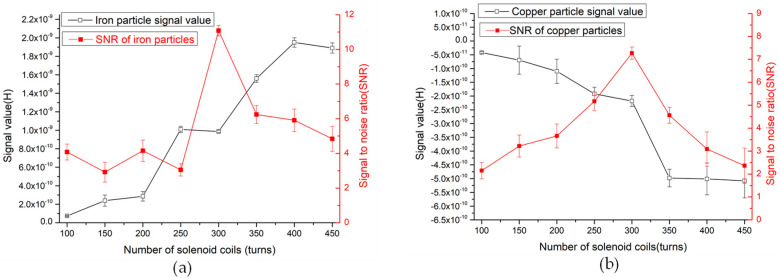
(**a**) Detection results of 40 μm iron particles by sensors with different turns; (**b**) detection results of 90μm copper particles by sensors with different turns.

The results in Figure 7 show that when detecting copper particles, the signal value increases as the number of coils turns increases. The signal of the 90μm copper particles detected by the 100-turn coil is −4.19 × 10^−11^. At 300 turns, the signal value increases to −2.18 × 10^−10^. At 450 turns, the signal value increases to 5.08 × 10^−10^. As the number of coils turns increases, the noise interference also changes. At 100–300 turns of the coil, the noise value gradually decreases, at 300 turns, the noise value is relatively low, and gradually increases at 300–450 turns. The SNR ratio of the copper particles calculated based on the signal value and noise value is shown in Figure 8b below.

Figure 8 is the result of the detection law of different turns sensors, which is more intuitive. As the number of turns increases, the detection signal value increases when detecting iron particles and copper particles. However, due to different noise values, the overall SNR value first increases and then decreases, and the SNR is the highest at 300 turns. Therefore, according to the results of this experiment, the 300-turn solenoid coil has the highest sensitivity, and the 300-turn coil is the design parameter of the sensor.

### 4.3. Discussion of Excitation Frequency

**Figure 9:**
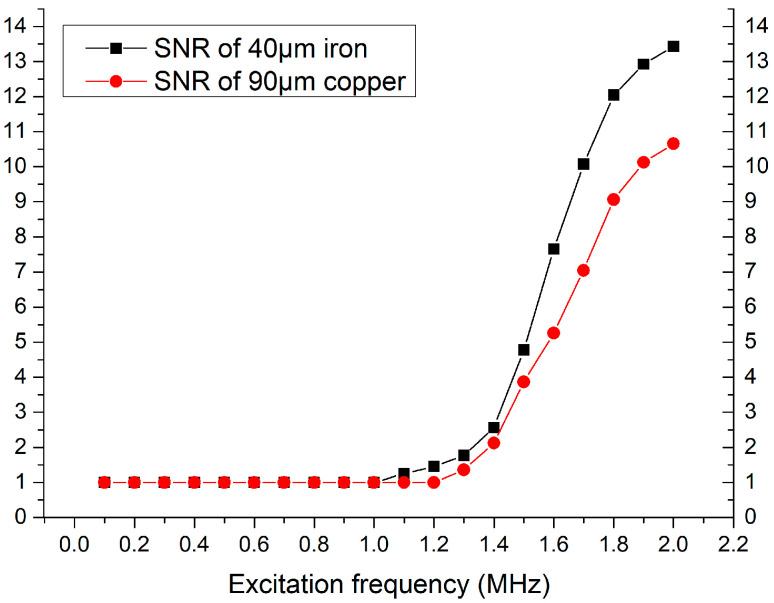
Frequency experiment results.

According to the above results, a 300-turn coil gives the best design and fabrication parameter of a 3-D solenoid, and experiments on frequency parameters are performed. In this experiment, we used the single variable method to control the number of turns of the coil. The 3-D solenoid is chosen at 300 turns and the frequency experimental results were measured using 40 μm iron and 90 μm copper particles, as shown in Figure 9.

According to the results in Figure 9, when the excitation frequency provided by the impedance analyzer is less than 1 MHz, the sensor has no detection effect on iron particles and copper particles, but when the excitation frequency is greater than 1 MHz, the detection SNRs increases as the excitation frequency increases. The maximum excitation frequency provided by impedance analysis is 2 MHz. At this time, when the sensor detects iron and copper particles, the maximum detection SNRs is obtained. Therefore, according to the experimental results in this section, we know that the optimal frequency for determining the sensor detection is 2 MHz.

### 4.4. Sensor Measures Multi-Size Contaminants

**Figure 10:**
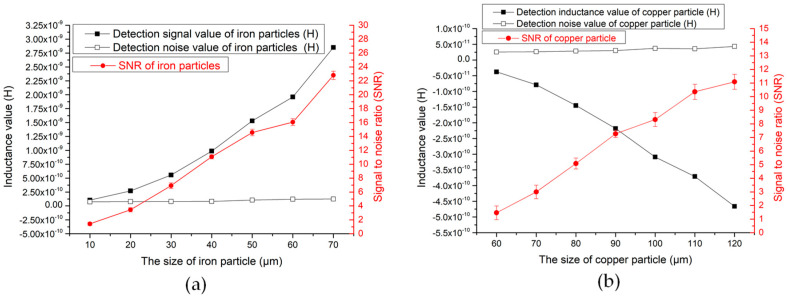
Sensor coil 300 turns, excitation frequency 2 MHz (**a**) average detection signal value and signal-to-noise ratios (SNRs) of iron particles of different sizes; (**b**) average detection signal value and SNRs of copper particles of different sizes.

According to the content of the previous section, when the number of coil turns is 300 and the excitation frequency is 2 MHz, the sensor has the best detection performance. Therefore, the sensor is used to detect ferromagnetic particles and non-ferromagnetic particles in different size ranges to further analyze the performance of the sensor. Before the experiment, iron particles are prepared, with sizes ranging from 10 to 70 μm, and copper particles are prepared, with sizes ranging from 60 to 120 μm. Then these particles are mixed with hydraulic oil separately, and the steps are the same as the previous experiment chapter. The mixed oil is put into the microinjection pump and tested for contaminants. After five experiments for particles of each size, the average value is calculated and the test results are shown in Figure 10.

When the sensor detects particles, the larger the particle size, the larger the detection signal value and the larger the SNR. When the detected noise value fluctuates, the SNR will also change with the fluctuation of the noise value. Figure 10a above is an example, the red curve in the figure is the SNR, and the white curve below is the noise value curve. When the noise value fluctuates at 50 μm and 60 μm particles, the SNR curve also fluctuates. This part calculates the SNR fluctuation error of the sensor in detecting particles of the same size, which can get the most accurate SNR value and ensure the stability of the sensor, as shown in Table 1.

**Figure 11:**
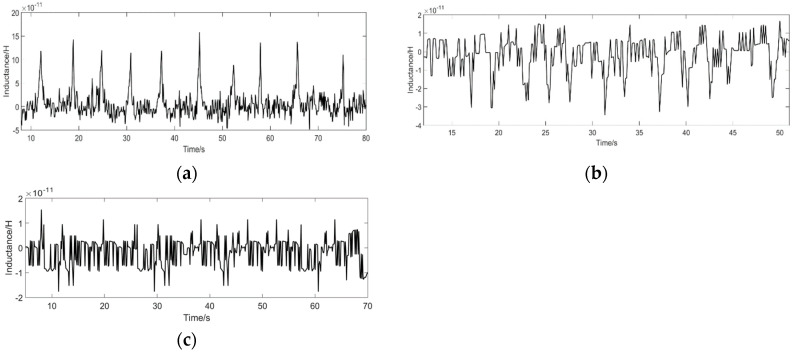
Sensor coil 300 turns, excitation frequency 2 MHz (**a**) detection signal value of 10 μm iron particles; (**b**) detection signal value of 60 μm copper particles; (**c**) test results of iron particles less than 10 μm and copper particles less than 60 μm, no signal value.

The sensor’s lower detection limit for iron particles is 10 μm, at this time the error value of the SNR is 0.22, and the SNR through calibration is 1.41 ± 0.22. The maximum error value of the SNR in the detection of 70 μm iron particles is 0.6, and the SNR is 22.8 ± 0.6 through the error calibration. When the size of the iron particles is less than 10 μm, the signal value of the iron particles that cannot be detected, the detection SNR is 1, and the detection signal is shown in Figure 11c. When the sensor detects copper particles, the lower limit of detection is 60 μm, and the SNR is 1.47 ± 0.2. When the size of the copper particles is 120 μm, the maximum error of the detection SNR is 0.56, and the calibration SNR is 11.09 ± 0.56.

It can be seen from Table 1 that the sensor is relatively stable in detecting the SNR. As the SNR value increases, the error value gradually increases, but the overall value is still relatively small. It can be proved that the stability of the sensor is high. At the same time, the lower detection limit of the sensor was tested, and it was determined that the sensor has a high detection accuracy, which can identify iron particles of 10 μm and copper particles of 60 μm, which is a new breakthrough in this type of sensor. The following figure shows the lower detection limit signal of iron particles and the lower detection limit signal value of copper particles. As well as the detection results of iron particles below 10 μm and copper particles below 60 μm, the signal value of the particles cannot be detected.

### 4.5. Throughput Experiments

**Figure 12:**
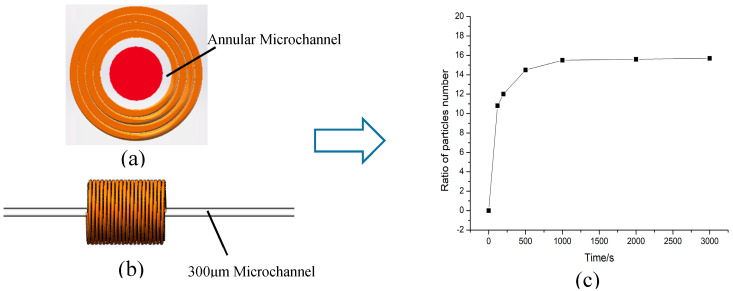
High-throughput comparison of microchannels. (**a**) The 300 μm annular microchannel designed in this paper; (**b**) 300 μm microchannel designed in the early stage; (**c**) Experimental results of high-throughput.

The main purpose of the annular microchannel designed in this paper is to increase the oil throughput, so the relevant to the laboratory is essential. Figure 12a shows the annular microchannel designed in this paper, the inner diameter of the annular microchannel is 300 μm; (b) is the 300 μm microchannel designed by the laboratory team in the early stage. According to the dimensions in Figure 1, the cross-sectional area of the annular microchannel is:

(13) $$S=\pi {\left(D_{1}+\frac{D_{2}}{2}\right)}^{2}-\pi \frac{D_{2}^{2}}{4}$$

Calculated through Formula (13), the flow rate has increased by 20 times. However, this result was not verified therefore, an experiment was conducted to confirm the result. In the experiment, the 300 μm microchannel produced previously by our team was selected [32] and the annular microchannels designed in this paper for comparison experiments. First, we determined that the oil flow speed in the two sensor microchannels are the same. Next, when selecting the 300 μm microchannel sensor experiment, the micro-injection pump flow rate was set to 30 μL/min. Since the theoretical flow rate of the annular microchannel is 20 times that of the 300 μm microchannel, the flow rate of the microinjection pump was set to 600 μL/min when performing the sensor experiment of the annular microchannel. Finally, the multiple of the flow increase by the ratio of the particles was determined.

The results in Figure 12c are experimental results of high throughput. The particle concentration in the oil is the same, so the experiment takes less time and has high reliability. From 0 to 1000 s, the ratio of the number of particles flowing through the two channels gradually increased, and after 1000 s, this ratio of the particles gradually stabilized. The particle ratio is maintained between ratio 15 and 16, which is less than the expected at ratio 20 of theoretical flow rate. The reason for these results may be the viscosity of the oil, the resistance of the glass tube to the fluid, or the increase in the number of particles.

## 5. Conclusions

In this paper, we designed an annular microchannel to detect metal abrasive particles in oil and combined it with a 3-D solenoid coil to make an inductive sensor. COMSOL simulation results prove that the structure of the annular microchannel is more suitable for 3-D solenoid type sensors, which makes the magnetic field more effective. Through the analysis of the experimental results, we found that when the 3-D solenoid has a 300-turn coil, the sensor has the largest detection of SNR. In the results of frequency characteristics, we found that the detection signal and SNR of the sensor increases as the excitation frequency increases. When the frequency is 2 MHz, the detection signal and SNR are the largest and the detection effect is at its best. We tested different sizes of particles, the detection limit for iron particles is 10 μm, and the detection limit for copper particles is 60 μm. At the same time, the SNR value and error of particles of different sizes are calculated, and the SNR of the sensor is corrected. Regarding high-throughput experiments, we found that the actual flux is slightly different from the theoretical value. This is due to the increase in the internal area of the annular microchannel and the increase in the number of particles flowing through it, which increases the resistance of the oil. This paper provides a new and effective method and technical support for the online detection of oil abrasive particles, which is of great significance for the prediction of the life-span and fault diagnosis of mechanical systems.

In future work, we will continue to improve the performance of this type of sensor. Then, we will collect a large amount of experimental data, simulate the test conditions, and establish a wear status database. At the same time, the sensor is completely separated from the laboratory equipment to achieve miniaturization and lightness. We will also design an online detection sensor that integrates sample delivery, detection and reading. According to the existing online database comparison, the attributes, types, sizes and quantities of pollutants in the oil are detected. This data could provide support for the operating state of mechanical equipment.

## Figures and Tables

**Table 1:** Calibration of SNR of different particles.

The Size of Iron Particle	SNR Value	SNR Error	Calibration SNR	The Size of Copper Particle	SNR Value	SNR Error	Calibration SNR
10 μm	1.41	0.22	1.41 ± 0.22	60 μm	1.47	0.2	1.47 ± 0.2
20 μm	3.45	0.3	3.45 ± 0.3	70 μm	3.01	0.31	3.01 ± 0.31
30 μm	6.92	0.4	6.92 ± 0.4	80 μm	5.09	0.34	5.09 ± 0.34
40 μm	11.08	0.3	11.08 ± 0.3	90 μm	7.27	0.27	7.27 ± 0.27
50 μm	14.57	0.47	14.57 ± 0.47	100 μm	8.32	0.31	8.32 ± 0.31
60 μm	16.06	0.5	16.06 ± 0.5	110 μm	10.36	0.5	10.36 ± 0.5
70 μm	22.8	0.6	22.8 ± 0.6	120 μm	11.09	0.53	11.09 ± 0.53
